# Opinions and beliefs held by Spanish teenagers regarding tobacco and alcohol consumption: a descriptive study

**DOI:** 10.1186/s12889-015-1417-y

**Published:** 2015-01-31

**Authors:** Roger Ruiz-Moral, Sara Palenzuela-Paniagua, Rosa Magallón-Botaya, Celia Jiménez-García, Jose Angel Fernández García, Luis Angel Pérula de Torres

**Affiliations:** Faculty of Medicine, University Francisco de Vitoria, Madrid, Spain; Health Center Otero, Ceuta, Spain; Health Center de Arrabal, Zaragoza, Spain; Sanitary District Cordoba and Guadalquivir, Maimonides Institute for Biomedical Research Córdoba (IMIBIC)/Reina Sofia University Hospital/University of Córdoba, Córdoba, Spain; Health Center Villarrubia, Maimonides Institute for Biomedical Research Córdoba (IMIBIC)/Reina Sofia University Hospital/University of Córdoba, Córdoba, Spain; Teaching Unit of Family and Community Medicine in Córdoba, Sanitary District Cordoba and Guadalquivir, Maimonides Institute for Biomedical Research Córdoba (IMIBIC)/Reina Sofia University Hospital/University of Córdoba, Córdoba, Spain; Escuela de Medicina, Edificio E, Universidad Francisco de Vitoria, Ctra. M-515 de Pozuelo-Majadahonda, Km. 1,800, 28223 Pozuelo de Alarcón, Madrid Spain

**Keywords:** Teenagers, Alcohol use, Cigarette smoking, Health behaviors

## Abstract

**Background:**

Preventive strategies are the most effective approach for dealing with issues of substance abuse, particularly in teenagers. Such strategies adapt well to this target population. Our objective was to reveal the opinions and beliefs held by teenagers about tobacco and alcohol as types of drugs, and their effects on health.

**Methods:**

In this cross-sectional study, participants completed a self-administered questionnaire based on the World Health Organization “Health Behaviour of School-aged Children” study. Our sample included 1,005 schoolchildren aged between 11 and 13 years, resident in the province of Córdoba in Spain. Descriptive and univariate analyses were performed using a chi-squared test.

**Results:**

Of respondents, 25% (95% confidence interval [CI]: 22.2–27.6%) and 61% (95% CI: 58.0–64.1%), respectively, did not consider tobacco or alcohol to be drugs. No relationship was found between tobacco and alcohol use, and the belief that these are drugs (*p* = 0.477 and *p* = 0.217, respectively). A total 98.2% of adolescents surveyed (95% CI: 97.3–99.1%) believed that tobacco causes physical damage, mainly to the lungs, heart, and to the developing fetus. Additionally, 92.4% (95% CI: 90.6–94.0%) believed that alcohol is detrimental to health and identified the liver as the organ most frequently damaged by alcohol consumption. The media was identified as the main source of information about these substances by 78.0% of respondents (95% CI: 75.4–80.6%).

**Conclusions:**

Teenagers possess an acceptable level of knowledge and information about the negative effects of tobacco and alcohol on health; however, many of them do not consider these substances to be drugs.

**Electronic supplementary material:**

The online version of this article (doi:10.1186/s12889-015-1417-y) contains supplementary material, which is available to authorized users.

## Background

Tobacco and alcohol are legal drugs that cause damage to human health and are addictive [1]. Tobacco contains nicotine, and various chemicals. Tar has been linked to cancer of the lungs and respiratory tract. Nicotine stimulates the heart and nervous system, thereby elevating heart rate and blood pressure. Alcohol is one of the most commonly used drugs, especially in Western countries [2], including Spain. Its effects, particularly those related to ethanol, although different in each individual, are well known. High-risk drinking, including frequent drinking and intoxication, is associated with adverse psychological, social and physical health effects. Alcohol use is one of the major risk factors for morbidity and mortality worldwide and is implicated in more than 60 different causes of ill health, constituting an enormous burden for individuals and societies [3].

Adolescence is the highest risk age group for the onset of use of these substances [4]. At this stage of life, social interaction mechanisms are significant in determining adolescent social behavior. The influence of peers and the mass media, the need for group affiliation, and the creation or consolidation one’s own identity are also important factors [5,6]. Adolescents and pre-adolescents often have a weak perception of the risks to which they expose themselves when they consume tobacco and alcohol. Unhealthy behaviors often stem from the pursuit of immediate pleasure or stress relief. However, the negative consequences of high-risk behavior are usually only evident in the long term. This is the reason first contact with alcohol or tobacco occurs very early on, and these become socially accepted behaviors early in life. Various studies have shown that the earlier experimentation with these substances, the greater the probability for their consumption to become problematic and the more difficult it becomes for users of tobacco and alcohol to give them up. In addition, contact with such substances can encourage or facilitate subsequent consumption of illegal drugs [7–11].

Most studies of the characteristics of tobacco and alcohol consumption have focused on adolescents aged 14 years and upwards. However, adolescents are engaging in high-risk habits at increasingly younger ages. Therefore, it is advisable to examine the attitudes and beliefs of youth in their preteen and early teen years so as to implement timely primary prevention and health promotion measures. This study examines the views of preadolescents and early adolescents toward alcohol and tobacco as drugs, their awareness about the harmful effects of these substances on the human body, and reveals the common ways in which they have access to information regarding these substances.

## Methods

### Design

We designed an observational, descriptive, cross-sectional (prevalence) study based on a health survey [12].

### Participants

The population in this study was children aged 11 to 13 years in the sixth grade of primary education (n = 8,944) in Córdoba Province, in the southern Andalusia region of Spain.

Selection was made by multistage random sampling in three phases: 1) stratified sampling by type of school (public or private) and geographic location—the city of Córdoba (urban area) or any provincial village (rural area); 2) by simple random sampling, we selected schools from a nominal list provided by the Andalusia Provincial Education Office; and 3) all students enrolled in the sixth grade at the selected schools were included by cluster sampling. A total of 954 schoolchildren was sampled, obtaining an accuracy level of ±3%, 5% alpha error, an anticipated proportion of 50% (situation of maximum uncertainty), and loss rate of 6%. Details on characteristics of the population and subpopulations sampled are shown in Table 1. After performing this sampling, the final sample size was 1005 subjects.

**Table 1:** **Study population and default sample by geographical area (rural, urban) and school type (public, private)**

Study population			
Province (rural area)		Capital (urban area)	
Public centers	Private centers	Public centers	Private centers
4,860 (88.07%)	579 (11.93%)	1,514 (44.20%)	1,911 (55.79%)
Subtotal: 5,518 (61.71%)		Subtotal: 3,429 (38.29%)	
Total population: 8,944			
Study sample			
517 (86.30%)	82 (13.70%)	226 (44.30%)	180 (55.70%)
Subtotal: 599 (59.6%)		Subtotal: 406 (41.4%)	
Total sample: 1005			

All schools in the geographic area studied were divided according to location and center type, and then listed in alphabetical order. We also arranged the number of students enrolled at each school as follows. We assumed that the average number of students per class was 35 and estimated a minimum number of schools and classrooms to be chosen at random. We used Epidat 3.1 statistical software for random selection.

#### Procedures

Data collection was done via a validated, self-administered, anonymous questionnaire that was based on that used by the World Health Organization study “Health Behaviour of School-aged Children” [13–15]. Initial contact with the directors of the selected schools was made by mail, and agreement about the dates for the survey was later reached by telephone. To avoid absences and subsequent selection bias, students were not told in advance about the survey. A letter with information regarding the study was sent to parents, and none objected to their child’s participation in the survey. Verbal consent was also requested of classroom teachers. Before beginning the survey, the researcher gave a short presentation to explain its purpose and instructions for completing the questionnaire, to ensure confidentiality. Verbal informed consent was requested from the students, and none refused to participate. Questionnaires were completed during a regular classroom lesson (45 to 60 minutes), with no teachers or tutors present so as to achieve greater confidentiality and truthfulness of the students’ responses.

The study was approved by the Research Ethics Committee of the Primary Health Care District of Córdoba, and permission was obtained from the Provincial Health Delegation and directors of all schools selected. Direct parental consent was not requested because this was an anonymous opinion survey, with no committed answers. In such cases, the Spanish educational and health authorities, as well as the Ethics and Clinic Investigation Committee, do not require direct parental consent.

### Measures

The questionnaire consisted of 57 questions grouped into seven blocks, each block preceded by an explanatory statement to inform students about what they would be asked. The present study focused on one block of questions (see Additional file 1), regarding the use of toxic substances. Questions included whether the students themselves had experimented with tobacco or alcohol, whether they considered these to be toxic substances, their opinions, knowledge and awareness of these toxic substances, the potential harmful effects of their use on human health, and the information they possessed.

### Statistical analysis

An inferential, descriptive statistical analysis was performed on qualitative variables, and absolute and relative frequencies were calculated. Among quantitative variables, central tendency, dispersion and position were measured, and 95% confidence intervals (95% CI) were calculated. The chi-squared test was used (with *p* < 0.05 considered statistically significant) to verify the relationship between consumption of tobacco or alcohol and the consideration or non consideration of both as drugs. Statistical analysis was carried out using Epidat 3.1 and SPSS 15.0 software.

## Results

A total of 1,005 surveys were completed, covering 28 schools in 19 municipalities in the province of Córdoba. Truancy on the day of the survey was 8.2% (n = 83). None of the students refused to take part in the study. Only seven surveys were not considered, because less than 75% of the questions had been answered. Average age was 11.45 (standard deviation ± 0.591), with range 11–13 years (95% CI: 11.42–11.49 years). The distribution of boys and girls was 52.8% and 47.2%, respectively. The respondents who admitted to smoking cigarettes occasionally was 9.5% (CI 95%: 7.7–11.4%). Average age at smoking onset was 10.38 ± 1.08, with range 8–13 years (95% CI: 10.08–10.67). The number of smokers increased with age (chi-squared 10,680; *p* = 0.005). We found 8.5% of boys and 4.6% of girls reported being current smokers (chi-squared 5.953, *p* = 0.015). No significant differences by location or type of school were found.

**Figure 1:**
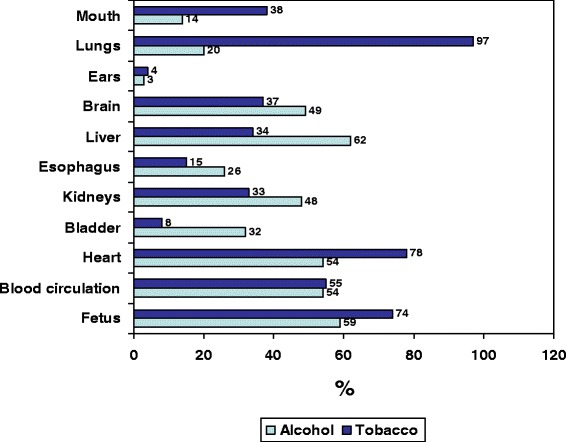
**Schoolchildren’s views on the damage caused by tobacco and alcohol.**

Nearly all (98.2%) respondents (95% CI: 97.3–99.1%) believed that tobacco use harms the human body, specifically the lungs (97%), heart (78%), and developing fetus (74%) (Figure 1). A total 18.7% of respondents (95% CI: 16.2–21.1%) reported having tried alcohol, with average onset age 11.54 ± 0.63 and range 11–13 years (95% CI: 11.45–11.63). A total 92.4% (95% CI: 90.6–94.0%) believed that alcohol was harmful. Boys (14.0%) drank more alcohol than girls (9.0%) (chi-squared 10.448; *p* = 0.005). The prevalence in alcohol consumption was higher among students of schools within the city than in rural areas (14.5% vs. 10.2%). According to students, the parts of the body most seriously affected by alcohol consumption are the liver (62%), circulatory system (54%), and heart (54%). They also highlighted harm to the fetus from alcohol use (59%) (Figure 1). No relationship was found between the use of tobacco or alcohol and the consideration of both as drugs (*p* = 0.477 and *p* = 0.217, respectively).

The percentage of students that claimed to have received information on drugs in general was 90.4%, and 78% indicated that the media was their main source of information, followed by their parents (57%) school tutors (51%), and health care professionals (31%). Schoolchildren from rural areas reported receiving less information about drugs (89.0%) than those at urban schools (93.0%) (chi-squared 3.835; *p* = 0.05). No statistical differences were found by age, sex, or public versus private school.

**Figure 2:**
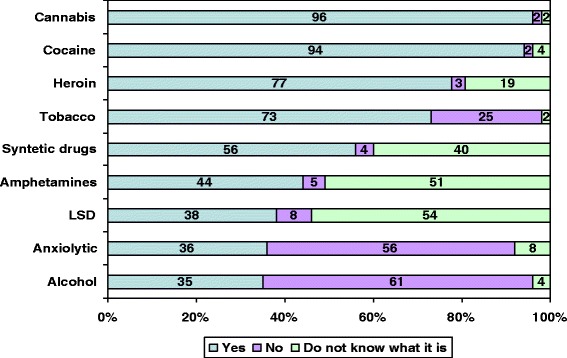
**Consideration of various substances as drugs.**

Figure 2 depicts the schoolchildren’s recognition of different toxic substances as drugs. Whereas 35% of students considered alcohol to be a drug and 73% considered tobacco to be a drug, cannabis was considered a drug by 96% of students. There were no significant differences in terms of age, sex, location or type of center in relation to students’ perception of these toxic substances as drugs.

## Discussion and conclusion

Although the vast majority of adolescents in this study were aware of tobacco and alcohol as being harmful to health and correctly identified the most commonly affected organs, up to one-third were unaware that tobacco is a drug and six of every ten students did not describe alcohol as such. This contrasts with their clear notions about drugs classified as illegal substances, such as cannabis, cocaine and heroin. The widespread misconception among preadolescents and adolescents that tobacco and alcohol are not drugs is very likely attributable to the legal nature of these substances for the adult population. The fact that adults consume both substances is seen by children as harmful to health, but they also consider this consumption “normal” within society. However, it is also necessary to consider the impact of advertising and the fact that families in particular, and society as a whole, are tolerant and permissive regarding the consumption of alcoholic beverages. For thousands of years, alcohol has been an integral part of the cultural and culinary heritage of western European countries, particularly those bordering the Mediterranean Sea [16].

Our findings suggest that measures implemented through mass media campaigns and other activities to make children in these age groups aware of the harmful effects of tobacco and alcohol on health have been effective. Nevertheless, such actions do not appear to have been adequate for teens and preteens to link these substances with the concept of drugs, despite having appropriate health information on the potential fatal consequences of consuming tobacco or alcohol. Therefore, it is important to bear in mind the paradox found within the phrase “nondrug-related harmful effect” when planning educational activities in various settings (schools, health centers, institutional campaigns in the media, and others), such that the danger involved in experimenting with tobacco and alcohol is not minimized in the minds of adolescents and preadolescents.

Information about drugs received by children seems to originate primarily from the media. It is striking that in only 31% of cases, this information has come from medical staff. Results from previous studies in the same area have shown similar results [17–20]. Without a doubt, the best information is not that from the mass media, because this is typically overly standardized, indiscriminate and biased [4,21–23]. The media provide information in the same way to both adults and children, and help establish patterns of behavior that are not always healthy. This is especially so for preteens, who are eager to experiment and imitate the attitudes and roles of adult behavior. Moreover, media advertising still encourages the use of certain drugs such as alcohol. Although advertising is regulated in most developed countries, children still receive contradictory messages, in favor of and against using certain substances. This may lead them to associate the use of legal drugs with fun and social maturity. Other people in children’s daily lives may also contribute to such associations with their permissive views on these drugs, and children will eventually imitate them to a greater or lesser extent [5,18,23,24]. According to a study in Spain [5], up to 20% of billboards at outdoor public locations near secondary schools showed advertising for alcoholic beverages or activities related to their consumption, although no advertising could be found for tobacco products. This fact can be explained by continuous anti-drug campaigns, “self-censorship” by the tobacco industry, and the introduction of regulations on tobacco advertising. These measures have almost completely eliminated tobacco outdoor advertising. The fact that cigarette smoking has been increasingly condemned and tobacco advertising more regulated, despite classification as a legal drug just like alcohol, clearly shows that the negative influence of advertising can be completely eradicated if there is a firm commitment to do so.

It is therefore necessary to promote children’s access to accurate information through other means and by people who are the most skilled, at least in theory, to inform them in the most effective and appropriate manner. It is clear that medical staff (particularly those who work in primary health care, such as family doctors, nurses, and psychologists) must take a leading role. Not only should these providers take advantage of adolescents’ visits to their surgeries and clinics (however infrequent such visits may be), these professionals should also take a more proactive approach by fostering and participating in, both as a group and individually, health education activities that are held in schools in their area. These activities can be done in coordination with teachers to support their work because, apart from the children’s own families, teachers are the closest everyday role model for teens and pre-teens. The specific training of teachers on this issue, together with implementation of health education campaigns for parents regarding identified needs and a community intervention program for teens supervised by interdisciplinary groups (health care staff, psychologists, educators), is viewed as the best strategy to prevent experimentation with and use of these drugs by young adolescents [5,25–27].

Certain studies have assessed the effectiveness of programs designed to increase awareness about drugs and strengthen preadolescents’ resources and self-esteem, to counteract the many messages they have received since early childhood to experiment with and consume tobacco and alcohol. These studies conclude that although preteen awareness is an intermediate variable when determining consumption, beneficial effects of the programs tend to dissipate with time and reinforcement activities are required. In addition, such programs have proven more effective if they are interactive and participatory, involve the intervention of peers as mediators, and begin before the transition from primary to secondary school [13,28].

### Limitations of the present study

This study was carried out in a medium-sized Spanish province, with its own sociocultural characteristics. Although these characteristics are very similar to those of the country as a whole, this particularity may limit extrapolation of the study results. Concerning possible selection bias, the sample was representative of the study population. With respect to the two controlled variables (rural/urban location and public/private schools), there was no difference between the study population and sample.

The method is based primarily on the “Health Behaviour of School-aged Children” project, sponsored since 1982 by the World Health Organization Regional Office for Europe [14,15]. In our study, we adhered as much as possible to this method to obtain comparable information and reduce or neutralize potential sources of information bias. The questionnaire used was validated by previous studies in different European countries. We also carried out a pilot study to analyze its reliability through a test-retest procedure, and were able to confirm a good level of reproducibility.

In addition, all surveys were administered by the same survey interviewer to avoid any observer bias. The interviewer was present in classrooms at all times to make necessary clarifications and ensure that the data collection procedure was as homogeneous as possible. The questionnaire was anonymous and students were repeatedly reassured that confidentiality was guaranteed, to ensure that they answered the questions as truthfully as possible.

## Conclusions

Preadolescents and early adolescents have an acceptable level of knowledge and information about the negative effects of tobacco and alcohol on health. Nevertheless, many do not recognize these substances as drugs. Information on drugs received by children seems to originate primarily from the mass media. It is necessary to further promote the role of health professionals and educators to inform and educate young people with respect to drug abuse; activities that are in keeping with their professional roles.

## Additional file

Additional file 1:
**La educación para la salud: tabaco y alcohol.**
