# Supplementary material for: Opinions and beliefs held by Spanish teenagers regarding tobacco and alcohol consumption: a descriptive study
Source: BMC Public Health. 2015 Jan 31;15:61. doi: 10.1186/s12889-015-1417-y (PMC4318511; doi:10.1186/s12889-015-1417-y)
Supplement: Additional file 1: — La educación para la salud: tabaco y alcohol. [file 12889_2015_1417_MOESM1_ESM.doc]

Additional file 1

1.--From the list below, mark with an X who you have received information about drugs from.

|  | Yes | No |
| --- | --- | --- |
| Parents | □ | □ |
| Brothers or sisters | □ | □ |
| Friends | □ | □ |
| School or high school teachers | □ | □ |
| Mass media (TV, radio, journals, newspapers…) | □ | □ |
| Doctor | □ | □ |
| No one has informed me | □ | |

2.-Do you smoke tobacco, if only occasionally?

□ Yes.

□ No.

3.-At what age did you smoke your first cigarette?

When I was __________________________ years old.

4. How often do you currently smoke?

□ Every day.

□ Several times a week, but not every day.

□ Weekends.

□ A few times a month, or rarely.

5. Do you think tobacco causes damage to the human body?

□ Yes.

□ No.

6.-From the list below, please mark with an X the parts or organs of the body that you think are damaged by the consumption of tobacco (you can answer more than one box).

□ Mouth.

□ Lungs.

□ Ears.

□ Brain.

□ Liver.

□ Esophagus.

□ Kidneys.

□ Urinary bladder.

□ Heart.

□ Blood.

□ Fetus in the womb.

□ Others (please indicate):___________________________________________.

7. Do you drink alcoholic drinks?

□ Yes.

□ No

8. At what age did you drink for the first time?

When I was __________________________ years old.

9. Have you ever been drunk?

□ Yes, once.

□ Yes, several times.

□ Yes, often.

□ No, never.

10. Do you think alcohol causes damage to the human body?

□ Yes.

□ No.

11. From the list below, please mark with an X the parts or organs of the body that you think are damaged by the consumption of alcohol (you can answer more than one box).

□ Mouth.

□ Lungs.

□ Ears.

□ Brain.

□ Liver.

□ Esophagus.

□ Kidneys.

□ Urinary bladder.

□ Heart.

□ Blood.

□ Fetus in the womb

□ Others (please indicate):___________________________________________.
